# Second-Order Nonlinearity of Graphene Quantum Dots Measured by Hyper-Rayleigh Scattering

**DOI:** 10.3390/ma16237376

**Published:** 2023-11-27

**Authors:** Manoel L. Silva-Neto, Renato Barbosa-Silva, Georges Boudebs, Cid B. de Araújo

**Affiliations:** 1 Programa de Pós-Graduação em Ciência de Materiais, Universidade Federal de Pernambuco, Recife 50670-901, PE, Brazil; manoel.neto@utoronto.ca; 2 Departamento de Física, Universidade Federal de Pernambuco, Recife 50670-901, PE, Brazilcid.araujo@ufpe.br (C.B.d.A.); 3 Univ Angers, LPHIA, SFR MATRIX, F-49000 Angers, France

**Keywords:** hyper-Rayleigh scattering, graphene quantum dots, second-harmonic generation

## Abstract

The first hyperpolarizability of graphene quantum dots (GQDs) suspended in water was determined using the hyper-Rayleigh scattering (HRS) technique. To the best of our knowledge, this is the first application of the HRS technique to characterize GQDs. Two commercial GQDs (Acqua-Cyan and Acqua-Green) with different compositions were studied. The HRS experiments were performed with an excitation laser at 1064 nm. The measured hyperpolarizabilities were $\left(1.0\pm 0.1\right)\times 10^{-27}\;esu$ and $\left(0.9\pm 0.1\right)\times 10^{-27}\;esu$ for Acqua-Cyan and Acqua-Green, respectively. The results were used to estimate the hyperpolarizability per nanosheet obtained by assuming that each GQD has five nanosheets with 0.3 nm thickness. The two-level model, used to calculate the static hyperpolarizability per nanosheet, provides values of $\left(2.4\pm 0.1\right)\times 10^{-28}\;esu\;$(Acqua-Cyan) and $\left(0.5\pm 0.1\right)\times 10^{-28}\;esu\;$(Aqua-Green). The origin of the nonlinearity is discussed on the basis of polarized resolved HRS experiments, and electric quadrupolar behavior with a strong dependence on surface effects. The nontoxic characteristics and order of magnitude indicate that these GQDs may be useful for biological microscopy imaging.

## 1. Introduction

The hyper-Rayleigh scattering (HRS) technique has been employed for several years to investigate the nonlinear optical properties of molecules in liquid suspensions as well as colloidal metal particles in aqueous solutions, nanocrystals in liquid suspensions, and various other materials. It entails directing a laser beam onto a sample and detecting the scattered light, that has double the frequency of the incident light. The intensity of the scattered light is contingent on the first hyperpolarizability of the sample molecular property (individual particles in our context), a measure of their ability to alter the polarization of light. In principle, the HRS technique provides a direct assessment of the magnitude of the first hyperpolarizability of molecules and nanoparticles that describes the second-order nonlinear optical (NLO) response. Substantial NLO activity is a crucial prerequisite for emerging technological photonic applications such as photonics and all-optical switching and optical parametric oscillators, which have intensified the quest for NLO-active materials. For example, previous research has underscored the remarkable NLO response in solutions of metallic nanometer-sized particles in solutions. The origin of this response lies in the one- or two-photon resonance of the incident laser light used in the experiments, coupled with the particles’ strong surface plasmon absorption. Graphene quantum dots (GQDs) are nanometer-size graphene segments that are small enough to exhibit exciton confinement and quantum size effects. Unlike graphene, they present a non-zero energy bandgap that is responsible for their characteristic electrical and optical properties. 

Generally, the optical absorption spectra of GQDs present a strong peak in the ultraviolet (≈230 nm)  due to the $\pi \to \pi^{*}$ excitation of the π  bonds of aromatic C=C and a weaker peak at ≈300 nm due to $n\to \pi^{*}$ transitions of C=O bonds [1,2]. The introduction of functional groups into the GQDs may lead to new absorption features and may change the GQDs’ optical properties [3,4]. Moreover, since they present molecule-like structures the bandgap can be tuned by changing the GQD size and by surface chemistry. Therefore, the optical properties of GQDs are dependent on the preparation method as well as the functional groups added to the graphene segments [3,4,5,6,7,8].

When compared to heavy-metal-based semiconductor quantum dots that cause concern for in vivo bioimaging, GQDs, due to their low toxicity [9,10] and strong two-photon induced fluorescence, have been exploited for biological applications by many authors [3,11,12]. Likewise, there are reported applications of GQDs for energy conversion, environmental monitoring, and electronic sensors for humidity detection, among other applications [3,4,7,13,14]. Recently, Qi et al. reported second harmonic generation (SHG) in boron doped-graphene quantum dots (B-GQD) and their application in stem cell imaging and tracking wound healing. These applications were achieved because the doping promoted the break of symmetry in the B-GQD allowing the SHG signal [15].

Although optical absorption, linear luminescence, second harmonic generation, and the third-order nonlinearity of GQDs have been studied [5,6,7,8,16,17,18], the second-order nonlinearity, associated with the first hyperpolarizability, β(ω), did not receive much attention. It is true that the experiments to be performed are not very easy to set up, such as measuring nonlinear refraction in a 4f setup (see for example [19]) to characterize the third-order NL response. Computational results were reported in [20,21,22] for some GQDs but no experimental result is available. Here, we want to estimate the order of magnitude of these coefficients knowing that the manufacturing methods relative to new materials, sometimes different from one lab to another, could influence slightly the result. The absorption spectra of certain DIY items, assumed to be identical, exhibit partial mismatches owing to the presence of significant residues (impurities) within the final solution. Hence, it proves beneficial to examine readily accessible, commercially available samples. Preparations employing hybrid manufacturing methods may yield slightly divergent results but should not alter the core conclusion in this context. 

In the present work, the hyper-Rayleigh scattering (HRS) technique [23,24] was employed for the first time to determine the effective first hyperpolarizability, $\langle \beta \left(2\omega \right)\rangle_{eff}$, of GQDs suspended in water. The HRS is an incoherent nonlinear process in which two photons with frequency ω are instantaneously combined to generate a new photon with frequency 2ω due to the interaction with a material. Unlike the two-photon induced luminescence process, the generation of second harmonic in the HRS process does not require on-resonance excitation of the medium to high energy levels. The HRS technique has been routinely used to study molecules [23,24,25,26,27], metal nanoparticles in liquid suspensions [28,29,30], and dielectric nanocrystals [31,32,33,34,35]. The first hyperpolarizabilities of molecules and nanoparticles can be determined as well as the origin of the incoherent second harmonic generation (SHG) signal detected.

Studying the second-order nonlinearity of graphene quantum dots is not only essential for advancing our fundamental understanding of these nanomaterials but also for unlocking their potential in a wide range of practical applications, from optoelectronics to material science and beyond. The origin of the second-order nonlinear effect arising from nanoparticles (NPs) with crystalline structure presenting inversion symmetry was discussed in [36,37,38,39]. Two important cases to consider are: NPs with noncentrosymmetric shape and NPs with centrosymmetric shape [39]. In the former, SHG is due to the induced electric dipole in the NP volume and in the interfaces. In the latter, the induced electric dipole does not occur because of the inversion symmetry in the crystalline structure of the NPs. Then, besides the contributions from the interfaces, an important mechanism of SHG is related to the field’s gradients occurring within the NP bulk [38,39]. However, it is difficult to synthesize NPs with perfect shape, and therefore, in real NPs, there is competition between the two main cases above mentioned.

In the present paper, we report values for the first hyperpolarizabilities of two different GQDs [40] investigated by measuring the intensity of the incoherent SHG signal upon excitation using a laser off-resonance with absorption transitions of the GQDs. Values of $\langle \beta \left(2\omega \right)\rangle_{eff}$ were determined by applying the HRS technique used for the calibration of para-nitroaniline (*p*-NA), which is the usual reference standard for HRS measurements. To determine the static hyperpolarizability per nanosheet, $\beta {\left(0\right)}^{NS}$, the classical two-level model [35,41] was applied in combination with the results for $\langle \beta \left(2\omega \right)\rangle_{eff}$ and considering that each GQD has five nanosheets with a thickness of 0.3 nm each. Studying the nonlinear optical properties of these materials can provide insights into their structure and functionality involving the interaction of light with nanoparticles at a nanoscale level.

## 2. Experimental Details

The experiments were performed with commercially available GQDs (Acqua-Green and Acqua-Cyan) purchased from STREM Chemical Incorporation (Kehl, Germany) [40]. Typically, these quantum dots, composed of graphene nanosheets, have diameters of about (5 ± 1) nm and average height of (1.5 ± 0.5) nm, corresponding to five graphene sheets. The GQD concentration in the present experiments was $\;3.4\times 10^{16}\;\mathrm{particles}/{\mathrm{cm}}^{3}$ for both samples that were suspended in water. Experiments were also performed with more diluted samples to verify their behavior with the laser intensity. The concentration of nanosheets per cm^3^ was estimated considering the data sheet provided by the manufacturer. The absorbance spectra of the samples were measured with a commercial spectrophotometer from 200 to 1500 nm. The photoluminescence (PL) spectra and their temporal evolution were measured using a fast photodetector with a time resolution of 1 ns.

**Figure 1:**
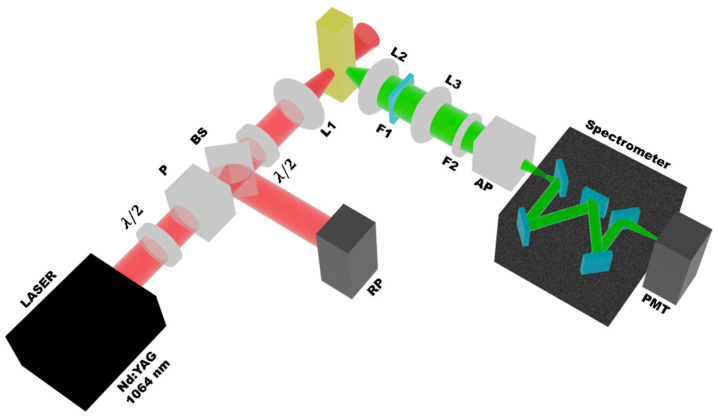
Experimental setup used for the hyper-Rayleigh scattering experiments. P is a Polarizer, BS is a Beam Splitter, RP is the Reference Photodetector, L1 is a focusing lens, L2 and L3 are collecting lenses, F2 is an interference filter, AP is an Analyzer Polarizer mounted in a rotation stage in a way that allows to set up the polarizer axis as Vertical (V) or Horizontal (H). PMT is a Photomultiplier Tube.

Figure 1 shows the setup used for the HRS experiments. The excitation source was a Q-switched Nd:YAG laser (1064 nm, 6 ns, 10 Hz). A bandpass filter (F1) was used to avoid scattered light from the fundamental beam. A half-wave plate (λ/2), a polarizer (P), a beam splitter (BS), and a reference photodetector (RP) were employed to control the incident laser intensity on the sample. A 5 cm focal length lens (L1) was used to focus the laser beam on the sample that was contained in a 1 cm long quartz cuvette. The HRS signal was collected perpendicularly to the laser beam direction by using two lenses (focal length: 5 cm) to focus the HRS signal on the photomultiplier (PMT) that was coupled to a spectrometer. This setup allowed for the analysis of the spectral content of the scattered light. Experiments were also performed using an interference filter (F2) centered at 532 nm with full width at half maximum (FWHM) of 8 nm. The photomultiplier was connected to an oscilloscope and a computer for data collection. For temporal analysis of the scattered light, the SHG signal was focused on a fast photodetector (response time: 1 ns) instead of the spectrometer/photomultiplier. For the polarization dependence measurements, another half-wave (λ/2) plate and another polarizer (AP) were inserted in the experimental setup shown in Figure 1 and the measurements were performed following the procedure of [28,29,30]. The half-wave plate used for polarization dependence measurements was inserted between the beam splitter (BS) and lenses L1. It is important to mention that the half-wave was coupled to a stepper motor controlled by a Labview-based program. For the measurements, the stepper motor is rotated in increments of 0–180 degrees. The polarizer (AP) was inserted between F2 and the spectrometer. During the measurement, the second polarizer is held in the vertical (V) or horizontal (H) polarization. Para-nitroaniline (*p*-NA) dissolved in methanol was used as a reference standard [23].

## 3. Results and Discussion

**Figure 2:**
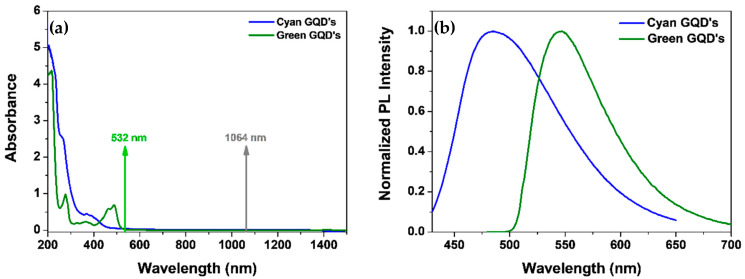
(**a**) Optical absorbance spectra; (**b**) One-photon induced photoluminescence spectra (excitation at 386 nm and 485 nm for Acqua-Cyan and Acqua-Green GQDs, respectively). The normalization factors for luminescence are $\;9.5\times 10^{5}$ for Acqua-Cyan GQDs and $1.7\times 10^{6}$ Acqua-Green GQDs. Samples concentrations: 3.4 $\times \;10^{16}\;\mathrm{particles}/{\mathrm{cm}}^{3}$.

Figure 2a shows the absorbance spectra of both GQDs samples. The absorption bands in the ultraviolet are associated with the $\pi \to \pi^{*}$ and $n\to \pi^{*}$ transitions [1,2]. The arrows in Figure 2a indicate the laser and the HRS wavelengths. Notice that, for both samples, the laser and its second harmonic are off-resonance with the absorption bands; therefore, we do not expect a PL signal superimposed on the HRS signal. Figure 2b shows the normalized PL spectra obtained by excitation at 386 nm for the Acqua-Cyan GQDs and 486 nm for the Acqua-Green GQDs using a commercial spectrofluorometer (Fluoromax HORIBA, Kyoto, Japan). The temporal decay of the PL signal with a maximum at 550 nm (Acqua-Green) and 480 nm (Acqua-Cyan), excited at 850 nm (via two-photon absorption), was fit by a single exponential with a decay time of 26 ns (Acqua-Green) and 19 ns (Acqua-Cyan).

**Figure 3:**
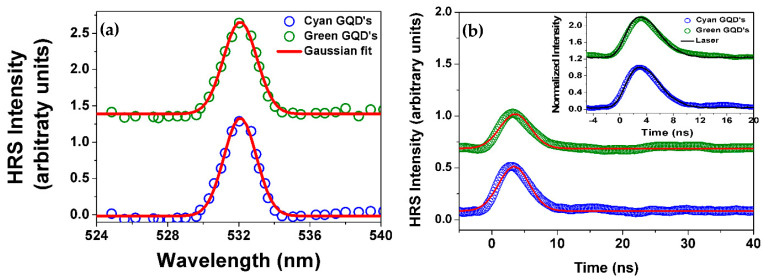
(**a**) Spectra of the incoherent second harmonic scattered light for excitation at 1064 nm. The continuous lines are Gaussian fits. (**b**) The green and blue circles show the temporal HRS data for both samples (single laser shot). The curves are shifted in the vertical direction to allow better visualization. The solid red lines are Gaussian fits to the data. The inset in (**b**) shows a comparison between the HRS signal and the excitation source. The green and blue circles in the inset show the temporal data for both samples and the black line illustrates the laser pulse temporal profile. The temporal response of the photodetector is 1 ns.

Figure 3 shows the HRS spectra and the temporal behavior of the HRS signal (obtained for a single laser shot). The solid red lines in Figure 3 are the Gaussian fit to the experimental data and the solid black lines on the inset of Figure 3b show the temporal evolution of the excitation laser. The duration for both GQDs was determined to be 4.6 ns from a Gaussian fit. Notice in Figure 3a that the spectra are centered at 532 nm, the second harmonic of the incident laser beam, with the linewidth limited by the spectrometer resolution. The temporal evolution of the HRS signal exhibited in the inset of Figure 3b follows the laser pulse corroborating the above statement that no long-lived luminescence overlaps with the HRS signal. The HRS is a parametric phenomenon, and the strong correlation between the temporal behavior of the signal and the excitation source supports this assertion. These measurements were performed with a laser peak intensity of $12\;\mathrm{GW}/{\mathrm{cm}}^{2}$, and the minimum intensity that allowed the detection of the HRS signal was $8.0\;\mathrm{GW}/{\mathrm{cm}}^{2}$. 

The HRS signal is due to the nonlinear scattering by the randomly oriented GQDs and water molecules. The scattered intensity, at the second harmonic frequency of the incident laser beam, is described by $I\;\left(2\omega \right)=g\;\underset{c}{\sum }N_{c}\langle \beta_{c}^{2}\left(2\omega \right)\rangle \;I^{2}\left(\omega \right)$, where I(ω) is the laser intensity, $\langle \beta_{c}^{2}\left(2\omega \right)\rangle =\langle \beta_{c}^{2}\left(2\omega ;\omega ;\omega \right)\rangle$ is the orientational average of the first hyperpolarizability and the sub-index represents the constituents of the suspension. The symbol 〈 〉 indicates orientational average. The factor g depends on the scattering geometry and contains information on the transformation of coordinates from the GQD to the laboratory reference system [31,32,33,34,35]. 

**Figure 4:**
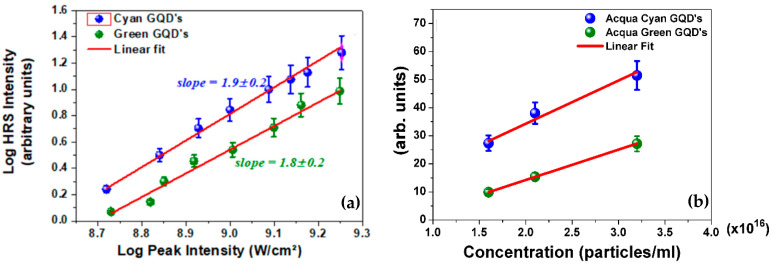
For better visibility, the data in blue (Acqua Cyan) have been shifted vertically. (**a**) HRS intensity signal, S(2ω)∝I (2ω), versus the laser intensity for both samples. The slopes of the straight lines were 1.9±0.1 (Acqua-Cyan) and 1.8±0.2 (Acqua-Green). (**b**) Dependence of S(2ω) with the concentration of GQDs per ml. The error bars represent 90% of confidence. The maximum peak intensity of the excitation source was 12 GW/cm^2^.

Figure 4a shows plots of the HRS signal, S(2ω)∝I (2ω), versus the incident laser intensity where a quadratic dependence with I (ω) was verified. Figure 4b shows the linear dependence of S(2ω) versus the GQDs concentration, $N_{GQD},$ which confirms the contribution of isolated GQDs to the HRS signal. Moreover, we recall that aggregation of GQDs would redshift the linear absorption spectrum, a behavior that was not observed for the various concentrations used. 

Since the samples consist of GQDs with different dimensions, the experiments provided effective values for the GQD hyperpolarizability, $\langle \beta^{2}\left(2\omega \right)\rangle_{eff}$. The external reference method [42], using *p*-NA dissolved in methanol as the reference standard, was applied to measure $\langle \beta \left(2\omega \right)\rangle_{eff}$ and the numerical values were obtained using the equation:

(1) $$\langle \beta^{2}\left(2\omega \right)\rangle_{eff}=\frac{S_{GQD}\left(2\omega \right)}{S_{pNA}\left(2\omega \right)}\left[\left\{\frac{N_{pNA}\langle \beta_{pNA}^{2}\rangle +N_{mtOH}\langle \beta_{mtOH}^{2}\rangle }{N_{GQD}}\right\}-\frac{N_{water}\langle \beta_{water}^{2}\rangle }{N_{GQD}}\right],$$

where mtOH refers to methanol, $\beta_{water}=0.087\times 10^{-30}\;esu$ [42], $N_{water}=5.5\times 10^{22}$ molecules/cm^3^, $\beta_{p-NA}=34\times 10^{-30}\;esu$ [22,23],$\;N_{p-NA}=1.0\times 10^{19}$ molecules/cm^3^, $\beta_{mtOH}=0.69\times 10^{-30}\;esu\;$ [23], $N_{mtOH}=1.50\times 10^{22}$ molecules/cm^3^. The concentration of GQDs was $N_{GQD}=3.4\times 10^{16}\;\text{particles}/{cm}^{3}$ for both samples that were suspended in water. Considering the results obtained for $S_{GQD}\left(2\omega \right)/S_{pNA}\left(2\omega \right)$ (see Figure 4a), the effective values of $\langle \beta \left(2\omega \right)\rangle_{eff}$ were $\left(1.0\pm 0.1\right)\times 10^{-27}\;esu$ and $\left(0.9\pm 0.1\right)\times 10^{-27}\;esu$ for Acqua-Cyan and Acqua-Green, respectively. The quantity $\langle \beta^{2}\left(2\omega \right)\rangle_{eff}$ is an effective value contributed by GQDs with different number of nanosheets. Consequently, we considered the following approach to estimate the hyperpolarizability per nanosheet, $\langle \beta^{2}\left(2\omega \right)\rangle_{NS}$. First we considered the distribution of GQDs sizes shown in the Appendix A; then, assuming the nanosheet thickness of 1.5 nm, and a Gaussian distribution of GQDs sizes represented by $P\left(n\right)\propto \exp\left(-n^{2}/n_{av}^{2}\right)$, where n is the number of GQD nanoparticles, we obtain 10≤n≤60 for Acqua-Cyan and 15≤n≤57 for Acqua Green. The value of $n_{av}$ is obtained from the AFM measurements given in the Appendix A. The value of $\langle \beta^{2}\left(2\omega \right)\rangle_{NS}$ is determined by $\langle \beta^{2}\left(2\omega \right)\rangle_{eff}=\langle \beta^{2}\left(2\omega \right)\rangle_{NS}\;\left\{\overset{n}{\underset{1}{\sum }}n\;P\left(n\right)/\overset{n}{\underset{1}{\sum }}P\left(n\right)\right\}$. Using these results, we could estimate the effective first hyperpolarizability per nanosheet, $\langle \beta \left(2\omega \right)\rangle_{eff}{}^{NS}\sim 10^{-28}\;esu$, considering that each GQDs have five nanosheets with 0.3 nm of thickness.

The two-level model [41,43] was used to relate $\langle \beta \left(2\omega \right)\rangle_{eff}{}^{NS}$ with the first static hyperpolarizability per nanosheet, $\beta {\left(0\right)}^{NS},$ in each GQD. First, it is necessary to estimate the static hyperpolarizability, β(0), for the samples. Then, considering the number of graphene sheets per GQD, $\beta {\left(0\right)}^{NS}$ is estimated. The following expression was obtained from [41,43].

(2) $$\beta \left(0\right)=\left(1-4\frac{\omega^{2}}{\omega_{eg}^{2}}\right)\left(1-\frac{\omega^{2}}{\omega_{eg}^{2}}\right)\langle \beta^{2}\left(2\omega \right)\rangle_{NS},$$

where $\omega_{eg}$ = 22,222 cm^−1^ (Acqua-Cyan) and $\omega_{eg}$ = 19,608 cm^−1^ (Acqua-Green). The frequency $\omega_{eg}$ correspond to the smallest band frequency in the absorption spectrum.

The results for $\beta {\left(0\right)}^{NS}$ are $\left(2.4\pm 0.1\right)\times 10^{-28}\;esu$ and $\left(0.5\pm 0.1\right)\times 10^{-28}\;esu$ for Acqua-Cyan and Acqua-Green, respectively. Notice that the value of $\langle \beta \left(2\omega \right)\rangle_{eff}{}^{NS}$ is approximately equal for both samples but the values of $\beta {\left(0\right)}^{NS}$ are different because of the different laser frequency detuning with respect to the first excited resonance frequency $\omega_{eg}$. 

**Figure 5:**
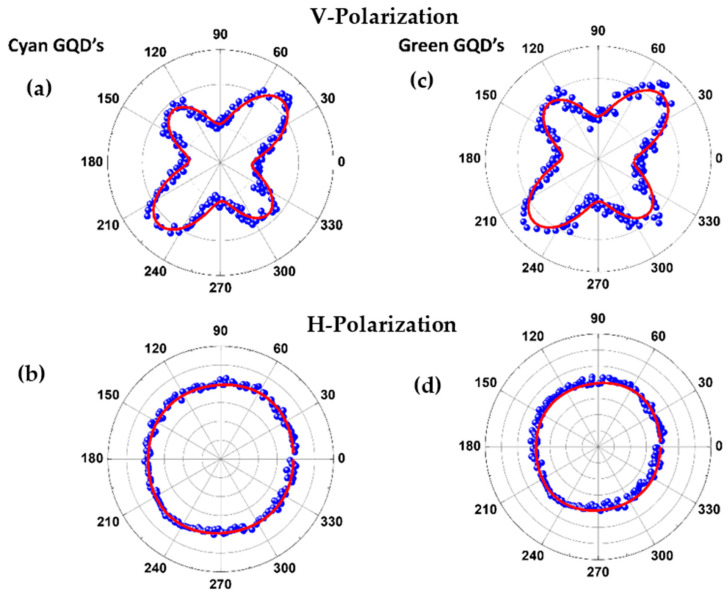
(**a**) Vertical polarization from Acqua-Cyan GQDs. (**b**) Horizontal polarization from Acqua-Cyan GQDs. (**c**) Vertical polarization from Acqua-Green GQDs. (**d**) Horizontal polarization from Acqua-Green GQDs. V and H polarizations are defined at the end of Section 2.

We recall that imaging measurements have been performed using GQDs exploiting their large PL, but SHG microscopy presents an important advantage with respect to PL experiments because lasers operating in a large infrared wavelength range can be used since no resonance with excited states is required. This is a benefit because one may select the laser wavelength according to the transparency window of the biological system of interest. Obviously, care must be taken not to damage living cells and the underlying nanoparticles with too high intensities, as can often happen with thin films irradiated with pulsed lasers in the picosecond regime [44]. To characterize the origin of the HRS signal, polarization-resolved experiments were performed following the usual procedure [28,29,30]. HRS is a technique for investigating the arrangement of molecules in nanostructures at the nanoscale. The SHG output of randomly placed and oriented signals is polarization-dependent due to the properties of nanostructures that are determined by the arrangement of molecules at the microscopic level within these elements. The HRS study reveals important charge transfer differences within supramolecular systems in suspension, depending on the extent of electronic polarization induced by the constituting molecules [45]. The laser beam was linearly polarized and the angle γ, between the optical field and the vertical direction, varied from 0 to 2π. The polarization of the HRS signal, either in the vertical direction or parallel to the beam propagation axis, was measured. At this point, due to the insertion of a half-wave plate and a polarizer (see Figure 1), the laser intensity was ≈12 GW/cm^2^. The results are shown in Figure 5 and, to fit the data, the following expression was used:

(3) $$I_{HRS}^{\Gamma }=a^{\Gamma }cos^{4}\gamma +b^{\Gamma }cos^{2}\gamma sin^{2}\gamma +c^{\Gamma }sin^{4}\gamma +d^{\Gamma }cos^{3}\gamma sin\gamma +e^{\Gamma }cos\gamma sin^{3}\gamma$$

where $I_{HRS}^{\Gamma }$ is the hyper—Rayleigh scattering intensity and Γ refers to the horizontal (H) or vertical (V) polarization. The coefficients $a^{\Gamma }$, $b^{\Gamma }$, $c^{\Gamma }$, $d^{\Gamma }$, $e^{\Gamma }$, were obtained from the fit. The measured values of $d^{\Gamma }$ and $e^{\Gamma }$ were too small compared to the other coefficients; therefore, these coefficients do not appear in Table 1. The multipolarity coefficients, $\varsigma^{\Gamma }=\left[1-(a^{\Gamma }+c^{\Gamma })/b^{\Gamma }\right]$, were also determined and their values are indicated in Table 1.

As mentioned before, the origin of the SHG rising from crystalline NPs presenting inversion symmetry is due to two factors: noncentrosymmetrical or centrosymmetrical shape. For nanoparticles presenting inversion symmetry and noncentrosymmetric shape, electric dipole surface second-harmonic response is allowed because of the surface of the particle, and the inversion symmetry of the bulk material is broken. In this case, retardation effects are not relevant for the SHG. On the other hand, for nanoparticles presenting inversion symmetry and centrosymmetrical shape, the retardation effects are important, and pure electric dipole contribution vanishes. The coefficient of multipolarity, $\varsigma^{V}$, is applied to distinguish both contributions [39]. The case for pure centrosymmetric shape requires $a^{V}=c^{V}=0$ and the case for the pure noncentrosymmetric shapes requires $b^{V}=a^{V}+c^{V}$. However, in real cases, the NPs are not perfect in shape or size. In this way, both the contribution of surface and volume may coexist. As one can see in Table 1, the values of $\varsigma^{V}$ for both samples suggest the dominant mechanism for harmonic generation is the field retardation in the NPs. This fact should be expected in our samples since there is no guarantee that the nanosheets are perfectly compacted and discordances should also occur between the sheets. As a result of such discordances, discontinuities in the electric field should occur in the NP volume and the quadrupole contributions become dominant. The negative value of the parameter $\varsigma^{V}$ can be interpreted as an effect from the nonspherical distribution of the GQD agglomerates [45].

Ongoing research is focused on assessing the toxicity of graphene-family nanoparticles, with specific attention to GQDs [46]. Factors such as particle size, synthesis methods, and chemical doping play a role in determining their toxicity, both in vivo and in terms of cytotoxicity [47]. Some researchers argue that GQDs, composed primarily of organic materials, exhibit low toxicity and high biocompatibility, providing an advantage over semiconductor quantum dots. In vitro studies using cell cultures have shown minimal bad effects of GQDs on human cell viability [48]. Fluorescence imaging, a non-radioactive method, allows the visualization of morphological details in various biological specimens, from living cells to animals. Notably, GQDs, unlike many semiconductor quantum dots containing heavy metals, consist of graphene lattices containing light elements. Because they are primarily made of carbon, the most abundant element in biological systems, GQDs are generally considered biocompatible. 

The analysis of beta per nanosheet is based on the number of nanosheets only, without considering their relative orientation and the fine structure of the scatterers. While our study sheds light on the polarization-dependent behavior of GQDs, further investigation is needed to elucidate the underlying mechanisms governing this phenomenon and to explore potential applications in detail. Future studies could delve into the specific factors influencing the polarization dependency, such as GQD size, shape, and surface functionalization. Additionally, exploring the potential integration of GQDs into practical devices and investigating their performance in real-world conditions would be instrumental.

## 4. Summary and Conclusions

In summary, we reported the incoherent second harmonic generation by aqueous suspensions of graphene quantum dots excited at 1064 nm. The hyper-Rayleigh scattering technique was applied for the determination of the first hyperpolarizability associated with the GQDs and the results indicate $\langle \beta \left(2\omega \right)\rangle_{eff}{}^{NS}\sim \;10^{-28}\;esu$ per nanosheet. The spectra of the scattered light (centered at the second harmonic frequency) as well as their fast temporal behavior prove that the signals detected are due to the second harmonic generation and not due to luminescence induced by two-photon absorption. From the hyper-Rayleigh scattering data, using a two-level model, we could determine the static hyperpolarizability per nanosheet, $\beta {\left(0\right)}^{NS}$. The values obtained were $\left(2.4\pm 0.1\right)\times 10^{-28}\;esu$ for Acqua-Cyan and $\left(0.5\pm 0.1\right)\times 10^{-28}\;esu$ for Acqua-Green. In the future, could we develop a straightforward theoretical model for predicting the second-order coefficients of optical nonlinearity, much like we can for third-order optical nonlinear coefficients? Theoretical forecasts for the nonlinear refractive index in infrared glasses are achievable [49]. The order of magnitude of the GQDs hyperpolarizabilities indicates that the two samples studied have potential for application in the microscopy of biological systems. Of course, the possible nontoxicity of the GQDs to living cells is also a relevant characteristic in comparison with many semiconductor quantum dots being used. 

## Figures and Tables

**Table 1:** Multipolarity parameters for both GQDs samples.

Sample Parameters	Cyan GQD V Polarization	Green GQD V Polarization
$a^{\Gamma }$	0.87	0.79
$b^{\Gamma }$	0.56	0.59
$c^{\Gamma }$	0.14	0.19
$\varsigma^{\Gamma }$	−0.81	−0.66

**Figure A1:**
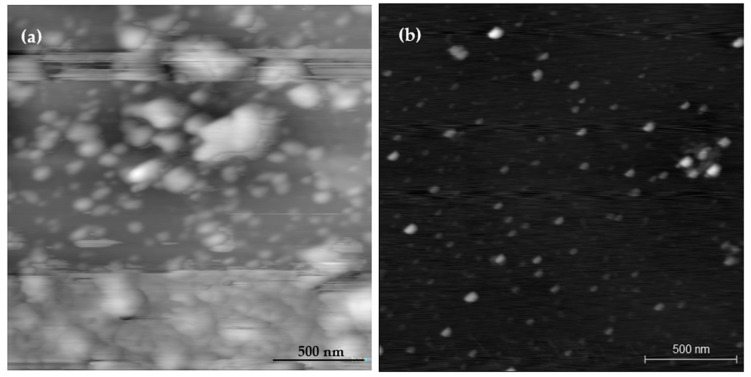
AFM images: (**a**) Acqua-Cyan GQDs; (**b**) Acqua-Green GQDs. The scale is 500 nm for both images.

**Figure A2:**
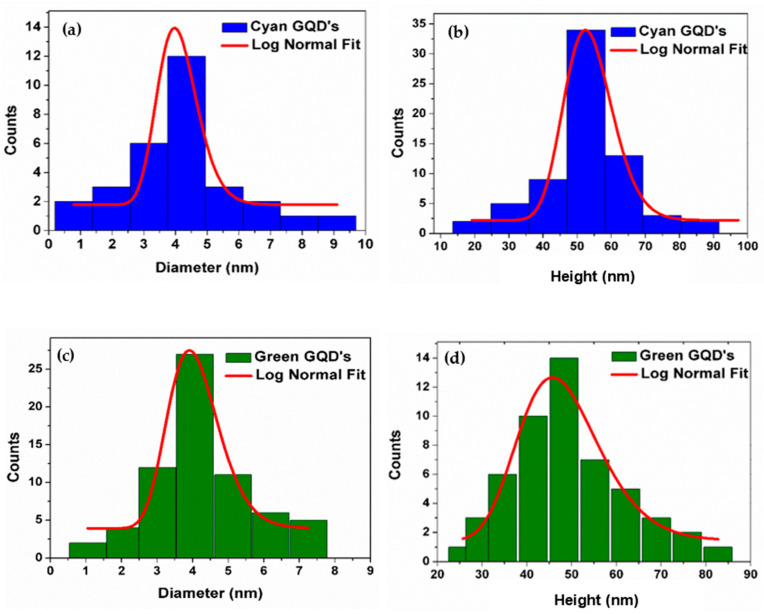
(**a**) Diameter and (**b**) Height of Acqua-Cyan GQDs; (**c**) Diameter and (**d**) Height of Acqua-Green GQDs.

**Figure A3:**
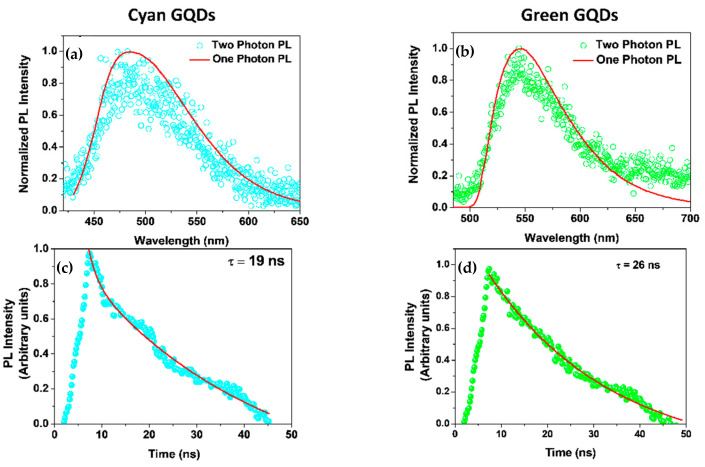
Normalized 1PL and 2PL Intensity (**a**) Acqua-Cyan GQDs and (**b**) Acqua Green GQDs. 2PL lifetime for (**c**) Acqua-Cyan GQDs and (**d**) Acqua-Green GQDs.

## Data Availability

Data underlying the results presented in this paper are not publicly available at this time but may be obtained from the authors upon reasonable request.

## Appendix A

### Characterization of the GQDs

The GQD dimensions were measured by Atomic Force Microscope (AFM) measurements. Figure A1 shows the AFM image for Acqua-Cyan and Acqua-Green GQDs.

From the AFM images, it was possible to make a histogram of the diameter and height of the GQDs, as shown below.

Figure A2 shows the histogram for the diameter and height of Acqua-Cyan and Acqua-Green GQDs. The log-normal fit indicates a maximum distribution for a diameter of 4 nm and height of 52 nm for Acqua-Cyan GQDs. For the Acqua-Green GQDs, the size distribution peaked for a diameter of 3.9 nm and a height of 46 nm.

Using the size distribution, we could calculate the concentration of GQDs per cm^3^. The value obtained was 0.9 × 10^15^ GQDs/cm^3^ for Acqua-Cyan and $1\times 10^{15}\;\mathrm{GQDs}/{\mathrm{cm}}^{3}$ for Acqua-Green.

Two-photon absorption-induced luminescence (2PL) with excitation at 850 nm was performed for both GQDs. The Figure A3a,b show the 1PL (solid lines) and 2PL (dots) spectra for both samples.

In Figure A3c,d we show the temporal behavior of 2PL, with a fluorescence lifetime of 19 ns for Acqua-Cyan GQDs and 26 ns for Green GQDs.
